# Modulating brain to improve goal-directed physical activity in older adults: a pilot study

**DOI:** 10.1093/geroni/igaf122.270

**Published:** 2025-12-31

**Authors:** On-Yee Lo, Levi Ask, Melike Kahya, Thomas Travison, Lewis Lipsitz, Brad Manor

**Affiliations:** Harvard Medical School/ Marcus, Hebrew SeniorLife, Boston, Massachusetts, United States; Clark University, Worcester, Massachusetts, United States; High Point University, High Point, North Carolina, United States; Hebrew SeniorLife & Harvard Medical School, Hebrew SeniorLife, Massachusetts, United States; Hebrew Senior Life, Boston, Massachusetts, United States; Hebrew SeniorLife/Harvard Medical School, Boston, Massachusetts, United States

## Abstract

Insufficient physical activity in older adults remains a global health issue. Several interrelated factors contributing to inactivity are linked to the prefrontal cortex. We conducted a pilot study to assess the feasibility, acceptability, and effects of combining transcranial direct current stimulation (tDCS) and behavior counseling to improve physical activity in older adults. Inactive older adults living in subsidized housing participated in this randomized controlled trial. Baseline physical activity (daily steps) was measured with a Fitbit for two weeks. Participants then received an eight-week intervention, including ten daily sessions of tDCS or sham stimulation during the first two weeks, along with four bi-weekly behavior sessions. Functional outcomes were assessed at baseline, post-stimulation, and after the entire intervention. Step counts were measured throughout the intervention and a 12-week retention period. Twenty-eight participants completed the study. Compliance was 97%, 93% and 92% for brain stimulation, behavior sessions, and follow-up assessments, respectively. Fitbit adherence was 96% and 71% during the intervention and retention periods. The tDCS arm, compared to sham, exhibited greater increase in average daily steps (p < 0.001). Participants increased 1179 (+22%) and 550 (+15%) steps/day from baseline in the tDCS and Sham arms, respectively. Motivation (p = 0.03) and self-reported walking performance (p = 0.02) were also improved in the tDCS arm compared to Sham. Combining tDCS and personalized behavior counseling to improve physical activity was feasible, acceptable, and appeared to be effective in a cohort of inactive older adults living within subsidized housing. Larger and more definitive studies are warranted.

